# LncRNA and mRNA expression profile of peripheral blood mononuclear cells in primary Sjögren’s syndrome patients

**DOI:** 10.1038/s41598-020-76701-2

**Published:** 2020-11-12

**Authors:** Yu Peng, Xuan Luo, Yingying Chen, Linyi Peng, Chuiwen Deng, Yunyun Fei, Wen Zhang, Yan Zhao

**Affiliations:** 1 Department of Rheumatology, Peking Union Medical College Hospital, Chinese Academy of Medical Sciences & Peking Union Medical College, Beijing, China; 2 Key Laboratory of Ministry of Health, National Clinical Research Center for Dermatologic and Immunologic Diseases (NCRC-DID), Beijing, China; 3 Department of Rheumatology, Clinical Immunology Center, Peking Union Medical College Hospital, Beijing, China

**Keywords:** Immunology, Pathogenesis, Rheumatology

## Abstract

The aim of this study was to elucidate the expression profile and the potential role of long non-coding RNA (LncRNA) in the peripheral blood mononuclear cells of primary Sjögren’s syndrome (pSS) patients. RNA-seq technology was used to detect the differentially expressed LncRNAs and mRNAs between five age-and sex-matched paired pSS patients and healthy control PBMCs. The selected LncRNAs were detected in the validation study by RT-qPCR in 16 paired pSS patients and healthy controls. The GO, KEGG, co-localization, and co-expression analysis were performed to enrich the potential gene functions and pathways. In this study, 44 out of 1772 LncRNAs and 1034 out of 15,424 mRNAs were expressed differentially in the PBMCs of pSS patients. LINC00426, TPTEP1-202, CYTOR, NRIR, and BISPR were validated as aberrantly expressed, and these LncRNAs strongly correlated with disease activity of pSS. GO and KEGG pathway analysis revealed the significant enrichment of biological processes, cellular components, and molecular function of the up and down-regulated mRNAs, which were mainly concentrated in the immune response and immune system processes. Co-localization and co-expression analysis also revealed that differentially expressed LncRNAs in the PBMCs of pSS were strongly correlated to the mRNA functioning associated with immune response and cell metastasis. Numerous LncRNAs and mRNAs were found differentially expressed in the PBMCs of pSS patients, especially NRIR and BISPR; they interacted with the co-localized and co-expressed mRNAs, which might participate in the pathogenesis of pSS through the NF-κB, JAK-STAT, and other signaling pathways that regulate cell metastasis.

## Introduction

Primary Sjögren’s syndrome (pSS) is a complex and heterogeneous systemic autoimmune disease with unknown etiology; and is characterized by the dysfunction of exocrine glands (mainly the salivary and lacrimal glands) and the infiltration of lymphocytes into the affected tissues^1,2^. Patients with pSS are commonly manifested by dry mouth and dry eyes, and other symptoms of organs involvement including lung, kidney and others^3,4^. pSS is a multifactorial disease resulting from genetic, environmental, and inflammatory factors; and aberrantly regulated innate and adaptive immunity pathways that were thought to play crucial roles in the disease^5^.

However, the inflammatory factors need precise regulation at the transcriptional and post-transcriptional levels. Recently, some studies have revealed that long non-coding RNA (LncRNA), which is transcribed by RNA polymerase II, consists of more than 200 nucleotides that cannot translate into proteins that play important roles in regulating inflammatory pathways^6,7^. LncRNA was widely expressed in organisms and participated in many important biological processes, including genomic imprinting, chromatin modification, intracellular signaling and so on^8^. Recent studies have revealed that LncRNAs are involved in the regulation of innate and adaptive immunity. LncRNA-COX2^9^, Lethe^10^, and linc1992^11^ can interact with RNA binding proteins (RBPs) and promote the release of corresponding cytokines, including IL-6, TNF-α, and IL-1β, which play a significant role in the regulation of innate immune responses. The expression of LncRNAs was highly specific at different stages of CD4^+^T cell differentiation, most of which could bind the transcription factors related to T cell functions (T-bet, GATA-3, and STAT4). LincR-Ccr2-5’AS^12^ and Lnc-MAF-4^13^ could regulate the specific Th2 differentiation. LncRNAs were recently reported to have important regulatory roles in autoimmune diseases (AID). NETA1 (activated MAPK signaling pathway through Toll like receptor (TLR) 4) was found to be elevated in systemic lupus erythematosus (SLE) patients^14^. Some inflammatory LncRNAs involved in the pathogenesis of autoimmune diseases were also found elevated in RA^15^, MS^16^, and other diverse autoimmune diseases. LncRNAs has significant role in the pathogenesis of autoimmune diseases. Some previous literatures have reported that LncRNAs had important roles in the pathogenesis (Immune regulation and cell metabolism) of labial gland of pSS patients^17,18^. While the different expression and function have not been found in pSS patients’ peripheral blood mononuclear cells (PBMCs) yet. Thus, in the present study, we used the RNA-seq technology to analyze the expression profile of LncRNAs and mRNAs in pSS patients’ PBMCs to investigate and further improve the potential roles of LncRNAs in the pathogenesis of pSS.

## Methods

### Patients and healthy controls

Twenty-one pSS patients and 21 age-sex matched healthy controls were enrolled in this study. All the pSS patients were recruited from the Peking Union Medical College Hospital and met the American-European consensus criteria for diagnosis^19^ and have not received any medication treatment. This study was approved by the Ethics Committee of Peking Union Medical College Hospital (Approval number JS-2038). This study was performed in accordance with the Declaration of Helsinki, all methods were performed in accordance with the relevant guidelines and regulations. Patients with other autoimmune diseases, active/severe infection or malignant diseases were excluded from the study. All the pSS patients and matched healthy controls in this study have signed the informed consent. The RNA-seq analysis was carried out on 5 pSS patient samples and 5 matched healthy controls while the following validation study consisted of 16 pSS patients and 16 matched healthy controls. After the patient enrolling stage, we enrolled 21 female pSS patients who met our inclusion criteria. Patient’s disease activity was determined by EULAR Sjögren’s syndrome disease activity index (ESSDAI)^20^, and serological examinations, including Immunoglobulin G (IgG), immunoglobulin A (IgA), immunoglobulin M (IgM), anti-SSA and SSB antibody, erythrocyte sedimentation rate (ESR), rheumatoid factor (RF), and so on, were performed for the study subjects. The detailed demographic and clinical features of patients and healthy controls are listed in Table 1.

**Table 1:** Demographic and clinical features of pSS patients and healthy controls in this study.

	pSS patients (n = 21)	Healthy controls (n = 21)
**Demographics**		
Age at onset	47.86 ± 11.55	45.86 ± 8.55
Sex (female)	21	21
Fever (n%)	2 (9.52%)	0
Joint pain (n%)	8 (38.10%)	0
ESSDAI (mean, S.D)	4.19 ± 2.54	N.D
**Laboratory examinations**		
Anti-SSA antibody (+/−)	18/3	Negative
Anti-SSB antibody (+/−)	8/13	Negative
Anti-Ro-52 antibody (+/−)	17/4	Negative
IgG (g/L, mean ± S.D)	21.59 ± 7.05	10.04 ± 2.72
IgA (g/L, mean ± S.D)	3.02 ± 1.14	2.06 ± 0.69
IgM (g/L, mean ± S.D)	1.62 ± 0.69	0.99 ± 0.37
C3 (g/L, mean ± S.D)	0.98 ± 0.23	1.04 ± 0.16
C4 (g/L, mean ± S.D)	0.19 ± 0.19	0.19 ± 0.07
ESR (mm/h, mean ± S.D)	28.05 ± 1.57	5.93 ± 3.13
RF (IU/mL, mean ± S.D)	126.51 ± 129.68	5.13 ± 4.06
CRP (mg/L, mean ± S.D)	2.35 ± 3.38	N.D

N.D. represented for “Not detect”.

### Sample processing

Peripheral blood (10 mL) was obtained from each subject and all the samples were collected in the ethylene diamine tetraacetic acid (EDTA) tubes. Peripheral blood mononuclear cells (PBMCs) were isolated using the Ficoll density gradient centrifugation and were counted using Cellometer Auto T4 (Nexcelom Bioscience, USA). Total RNA was extracted using TRIzol reagent (Invitrogen, Carlsbad, CA, USA) and the concentration of total RNA was measured by NanoDrop2000c spectrophotometer (NanoDrop Technologies, Wilmington, DE, USA). Then, the total RNA was reverse transcribed into complementary DNA (cDNA) using PrimeScript RT Master Mix (TaKaRa, Dalian, China) and was stored at – 80 ℃ for the following study.

### Library preparation, LncRNA sequencing

A total amount of 20 ng RNA per sample was used as input material for the RNA sample preparations. Firstly, ribosomal RNA was removed by Epicentre Ribo-zero rRNA Removal Kit (Epicentre, USA), and rRNA free residues was cleaned up by ethanol precipitation. Subsequently, sequencing libraries were generated using the rRNA-depleted RNA by NEBNext Directional RNA Library Prep Kit for Illumina (NEB, USA) following manufacturer’s recommendations. Then, we used the Qubit RNA Assay Kit and Qubit 2.0 Flurometer (Life Technologies, CA, USA) to quantify RNAs. RNA integrity was assessed using the RNA Mano 6000 Assay Kit of the Bioanalyzer 2100 system (Agilent Technologies, CA, USA).

### Clustering and sequencing

The clustering of the index-coded samples was performed on a cBot Cluster Generation System using TruSeq PE Cluster Kit v3-cBot-HS (Illumina, USA) according to the manufacturer’s instructions. After cluster generation, the libraries were sequenced on an Illumina Hiseq 2500 platform and 125 bp paired-end reads were generated.

### Quality control

Raw data (raw reads) of fastq format were firstly processed through in-house perl scripts. In this step, clean data (clean reads) were obtained by removing reads containing adapter, reads on containing poly-N and low-quality reads from raw data. At the same time, Q20, Q30 and GC content of the clean data were calculated. And all the down stream analyses were based on the high-quality clean data.

### Mapping to reference genome

Reference genome and gene model annotation files were downloaded from genome website directly. Index of reference genome was built using HISAT2 v2.0.4 and paired-end clean reads were aligned to the reference genome using HISAT2 v2.0.4.

### Transcriptome assembly

The mapped reads of each sample were assembled by StringTie v1.3.3 in a reference-based approach. String tie used a novel network flow algorithm as well as an optional de novo assembly step to assemble and quantitate full-length transcripts representing multiple splice variants for each gene locus.

### Differentially expressed LncRNAs

Differentially expressed LncRNA were analyzed by DESeq package based on the negative binomial distribution test. A threshold FDR < 0.05 and |log_2_Fold change|≥ 1.

### Real-time quantitative polymerase chain reaction (RT-qPCR)

The stored cDNAs were processed by TB Green Premix Taq II kit (TaKaRa, Dalian, China) and 7900 HT Fast Real-Time PCR System (ABI, Foster City, CA, USA). SYBR Green Buffer, other reagents in this kit, and the cDNAs were in a total volume of 10 uL and the RT-qPCR was performed in 384-well microplates. The conditions we used to select LncRNAs were: (a) The fold change of the LncRNAs must be greater than 1.5 fold compared with healthy controls and also P-value must be less than 0.05; (b) Genes with LncRNA-mRNA repeated seruences and LncRNAs which could not find information in databases were not selected in the RT-qPCR validation study. And based on the results of the RNA-seq, we selected 11 differentially expressed LncRNAs meeting the above conditions for validation study. The selected LncRNAs’ primer sequences were listed in supplementary Table 1. The expression of each LncRNA was represented as fold changes using the ^ΔΔ^Ct method to obtain RT-qPCR results.

### GO and KEGG enrichment analysis

Gene ontology (GO) enrichment analysis of differentially expressed genes or LncRNA target genes were implemented by the GOseq R package, with the gene length bias being corrected. GO terms with corrected P-value less than 0.05 were considered significantly enriched by differentially expressed genes. KEGG was a database resource for understanding high-level functions and utilities of the biological system, and KOBAS software was used to test the statistical enrichment of differential expression genes or LncRNA target genes in KEGG pathways^21,22^.

### Co-expression and co-location analysis

Cis role is LncRNA acting on neighboring target genes. We searched coding genes 10k/100k upstream and downstream of LncRNA, found the adjacent mRNAs and then analyzed their function next. Trans role is LncRNA to identify each other by the expression level. We calculated the expressed correlation between LncRNAs with R function to find out the co-expressed mRNAs and then predicted the potential functions of the LncRNAs in pSS.

### Statistical analysis

The results of the RT-qRCR were verified through t-test if satisfied normality distribution or through Nonparametric test if did not satisfy normality distribution. And the correlation between clinical features and validated LncRNAs were tested through Spearman’s correlation tests. All the data were analyzed using R Statistical Package (version 3.6.3; https://www.r-project.org). A P-value (two-tailed) < 0.05 was thought to be statistically significant.

## Results

### Differentially expressed LncRNAs and mRNAs in PBMCS of pSS patients

**Figure 1:**
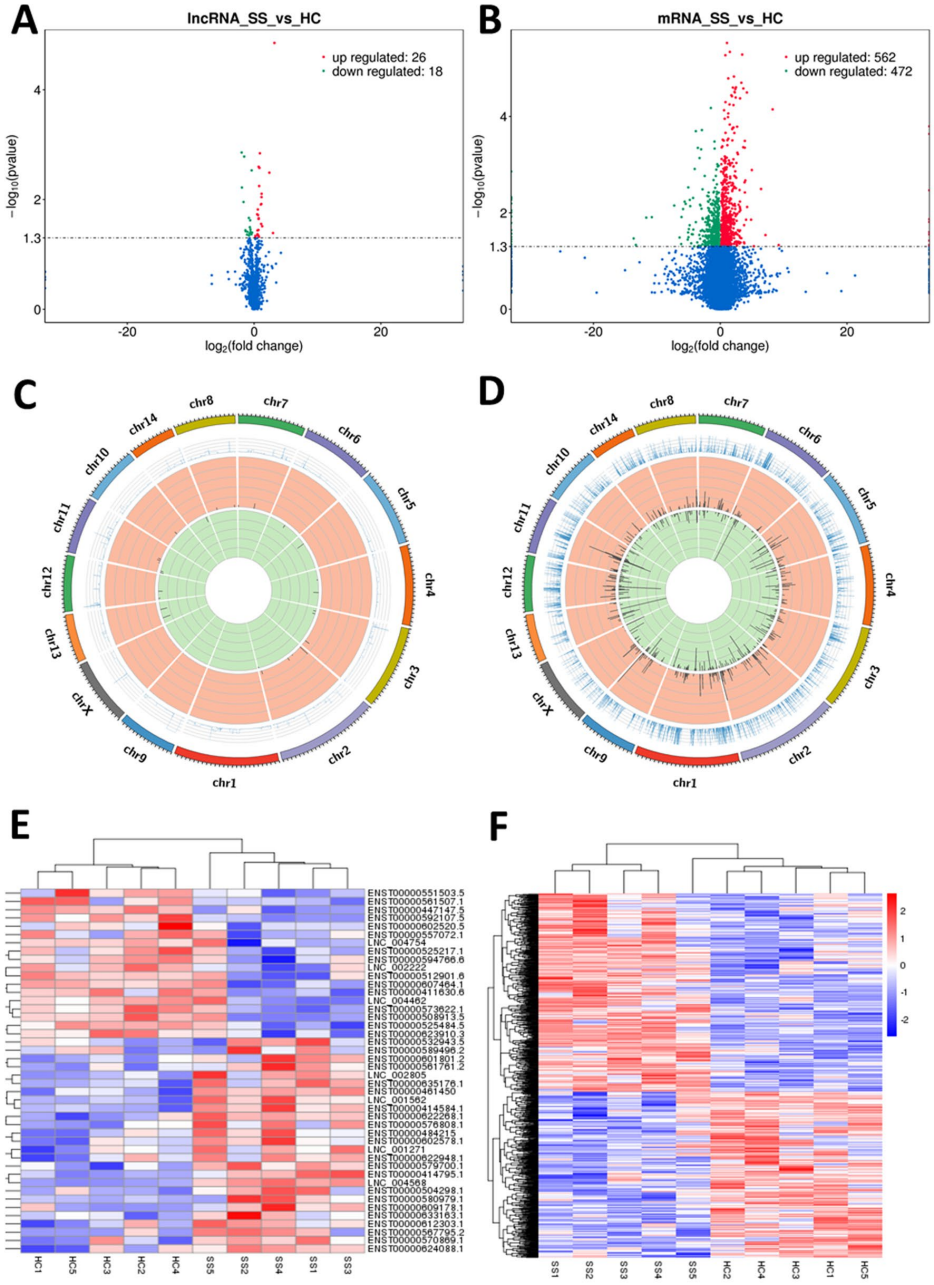
The LncRNA and mRNA expression profile of PBMCs in pSS patients. (**A**,**B**) The volcano plots for the differentially expressed LncRNAs and mRNAs between pSS patients and matched healthy controls, red stands for up-regulated and green stands for down-regulated. (**C**,**D**) The distribution of differentially expressed LncRNAs and mRNAs on chromosomes. (**E**,**F**) The heat maps for the hierarchical clustering of differentially expressed LncRNAs and mRNAs, red stands for up-regulated and blue stands for down-regulated. (R Statistical Package (version 3.6.3; https://www.r-project.org) was used to analyze the data of this figure and produce the figure).

Five pSS patients and 5 sex-age matched healthy controls participated in the RNA sequencing study. The volcano plot (Fig. 1A,B) showed the aberrantly expressed LncRNAs and mRNAs in pSS patients as compared to the healthy controls. Among all the 1772 tested LncRNAs, 44 LncRNAs were expressed aberrantly as found in this study, including 26 up-regulated and 18 down-regulated, and the top 10 up-regulated and down-regulated LncRNAs are listed in Table 2. Out of 15,424 tested mRNAs in this study, a total of 1034 mRNAs were found to express aberrantly, including 562 up-regulated and 472 down-regulated. The distribution of these differentially expressed LncRNAs and mRNAs was shown in Fig. 1C,D. We did not observe any special enrichment of the differentially expressed LncRNAs or mRNAs on the specific chromosomes. The hierarchical clustering analysis (Fig. 1E,F) showed distinct expression signatures for both LncRNAs and mRNAs. All the RNA-seq data were stored in the GEO database (Accession number GSE145065).

**Table 2:** The Top 10 up-regulated and down-regulated LncRNAs of PBMCs in pSS patients that are significantly and differentially expressed.

Up-regulated			Down-regulated		
Gene ID	Fold change	P-value	Gene ID	Fold change	P-value
XLOC_167004	9.25	< 0.001***	ENSG00000238121.5	− 3.92	0.001**
ENSG00000260114.2	7.89	0.041*	ENSG00000215068.7	− 3.78	0.006**
ENSG00000225964.5	5.28	0.003**	XLOC_078623	− 3.18	0.011*
ENSG00000272666.1	2.41	0.031*	ENSG00000100181.21	− 2.95	0.002**
ENSG00000265479.5	2.26	0.008**	ENSG00000246223.8	− 2.58	0.036*
XLOC_102167	2.24	0.009**	ENSG00000269893.6	− 2.51	0.038*
ENSG00000282851.1	2.20	0.012*	ENSG00000258511.1	− 2.17	0.044*
ENSG00000243398	2.16	0.027*	ENSG00000257261.5	− 2.07	0.039*
ENSG00000262979.1	2.08	0.012*	ENSG00000255026.1	− 1.78	0.022*
ENSG00000277999.1	1.88	0.001**	ENSG00000203875.10	− 1.74	0.039*

Gene ID were the gene in which the transcript resides.

*P-value < 0.05, **P-value < 0.01, ***P-value < 0.001.

### The results of RT-qPCR of 11 selected LncRNAs and the correlation between differentially expressed LncRNAs and clinical features

**Figure 2:**
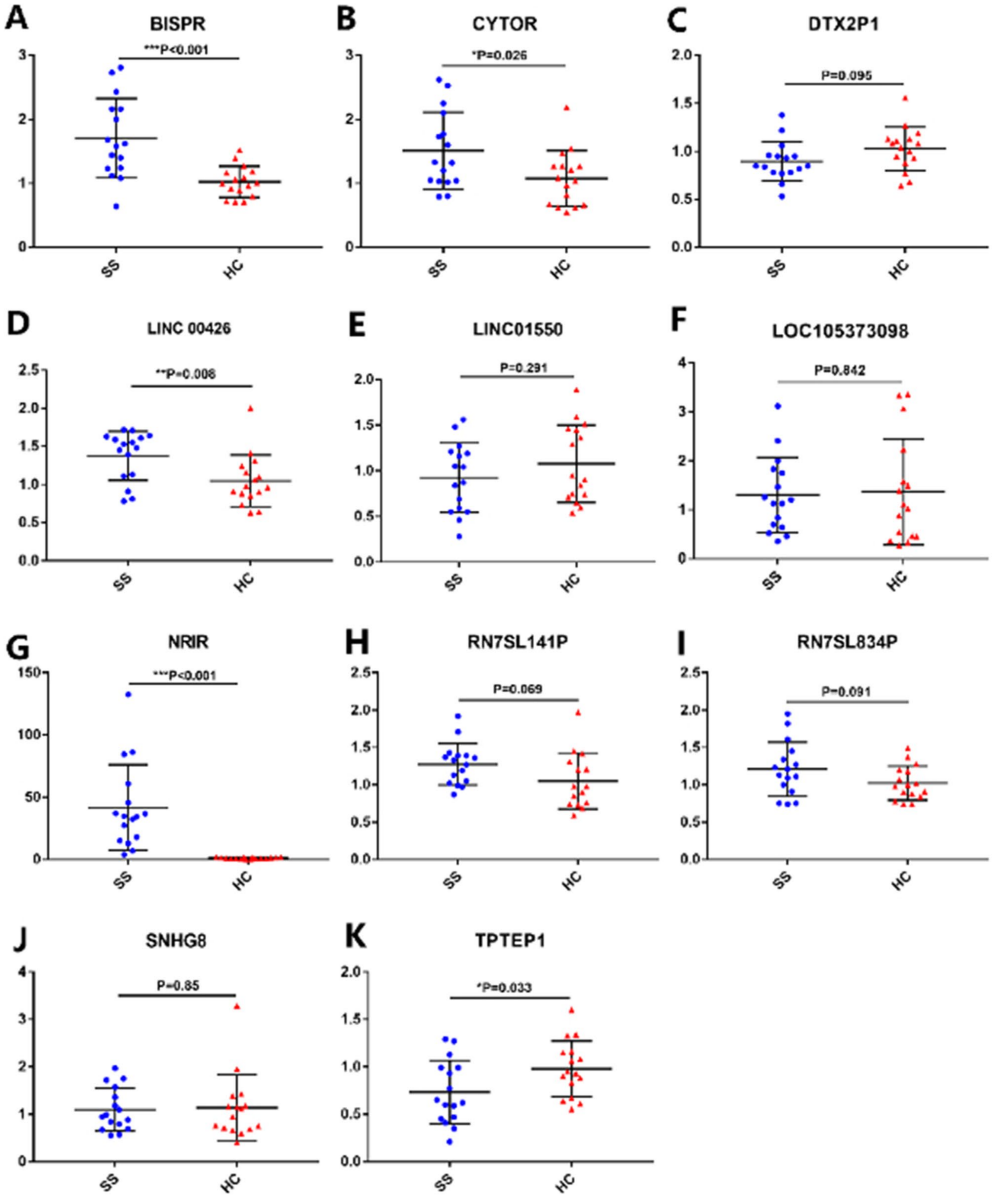
(**A–K**) The results of the validation study for the selected LncRNAs. BISPR (**A**), CYTOR-235 (**B**), DTX2P1-UPK3BP1-PMS2P11 (**C**), LINC00426 (**D**), LINC01550 (**E**), LOC105373098 (**F**), NRIR (**G**), RN7SL141P (**H**), RN7SL834P (**I**), SNHG8 (**J**), TPTEP1-202 (**K**) were selected for the validation study. Only BISPR (**A**), CYTOR (**B**), LINC00426 (**D**), NRIR (**G**), TPTEP1-202 (**K**) were significantly and differentially expressed.

**Figure 3:**
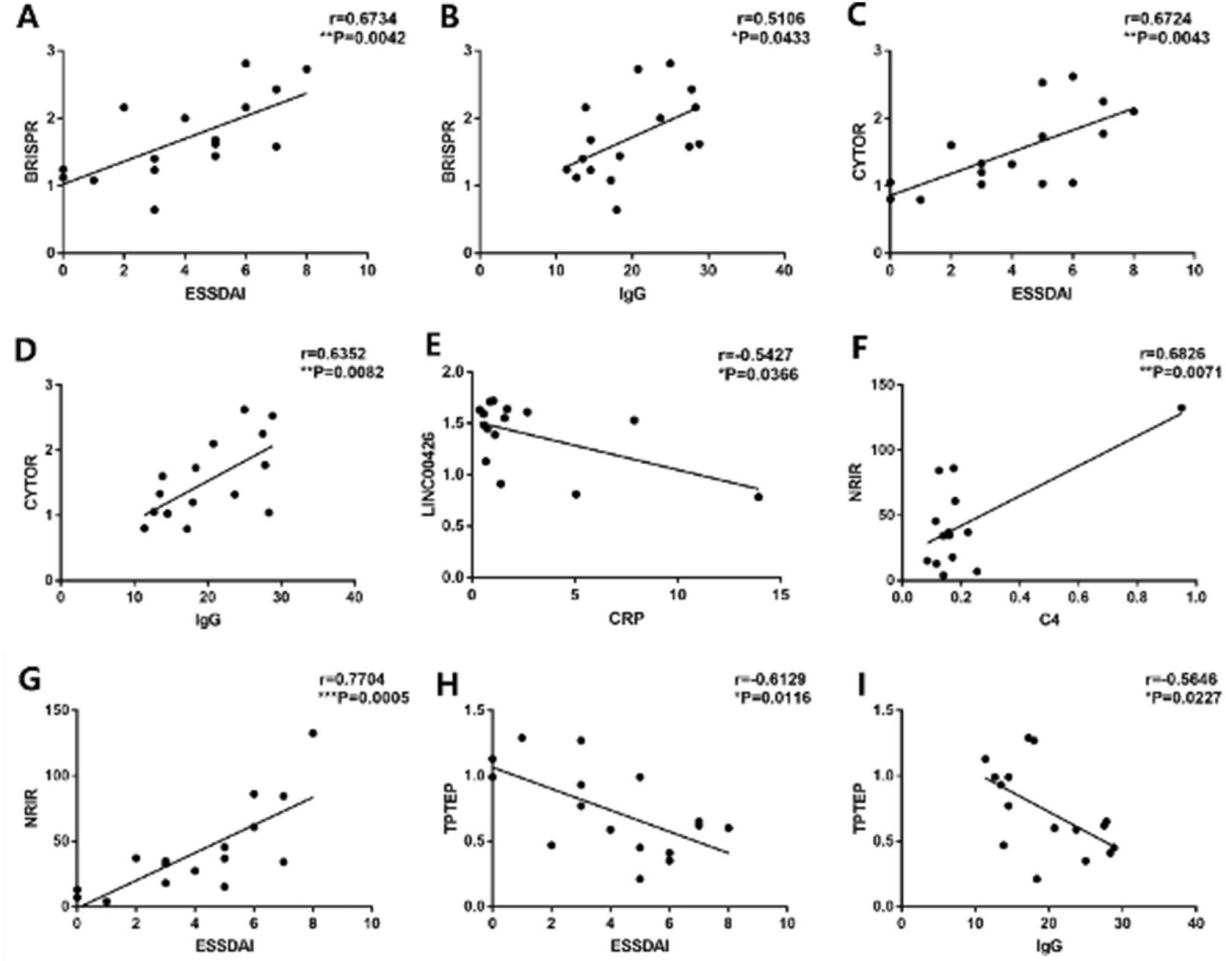
The correlations between the differentially expressed LncRNAs and patients’ clinical features. (**A**,**B**) The significant correlation between BISPR and ESSDAI, IgG. (**C**,**D**) The significant correlation between CYTOR and ESSDAI and IgG. (**E**) The significant correlation between LINC00426 and CRP. (**F**,**G**) The significant correlation between NRIR and ESSDAI, C4. (**H**,**I**) The significant correlation between TPTEP1-202 and ESSDAI, IgG.

Sixteen pSS patients and 16 matched healthy controls were involved at this stage. Eleven significantly differentially expressed LncRNAs were chosen for validation (the selection condition and primer sequences were listed above). Among the 11 selected LncRNAs, we conformed 5 LncRNAs differentially expressed through the RT-qPCR (Fig. 2), including BISPR (Fig. 2A, P < 0.001), CYTOR (Fig. 2B, P = 0.026), LINC00426 (Fig. 2D, P = 0.008), NRIR (Fig. 2G, P < 0.001), and TPTEP1-202 (Fig. 2K, P = 0.033). It was noted that NRIR and BISPR were significantly up-regulation in pSS patients, which were almost absent to lowly expressed in healthy controls but highly expressed in pSS patients. Then we analyzed the correlation between the differentially expressed LncRNAs and pSS patients’ clinical features. The significant correlation between LncRNAs and patient’s clinical features are shown in Fig. 3. BISPR and CYTOR were positively correlated with ESSDAI (r = 0.6734, P = 0.0042 with BISPR and r = 0.6724, P = 0.0043 with CYTOR) and serum IgG (r = 0.5106, P = 0.0433 with BISPR and r = 0.6352, P = 0.0082 with CYTOR). LINC00426 was negatively correlated with C-reactive protein (CRP) (r =  − 0.5427, P = 0.0366). NRIR was positively correlated with ESSDAI (r = 0.7704, P = 0.0005) and C4 (r = 0.6826, P = 0.0071). TPTEP1-202 was negatively correlated with ESSDAI (r =  − 0.6129, P = 0.0116) and IgG (r =  − 0.5646, P = 0.0227), while the correlation between NRIR and C4, LINC00426 and CRP seemed to be driven by some particular high outliers (Fig. 3E,F), thus LncRNA-NRIR and LINC00426 only had the trend of positive correlation with C4 and CRP, these correlation were still needed to be further tested in a lager sample size study.

### Co-localization and co-expression between differentially expressed LncRNAs and mRNAs

**Figure 4:**
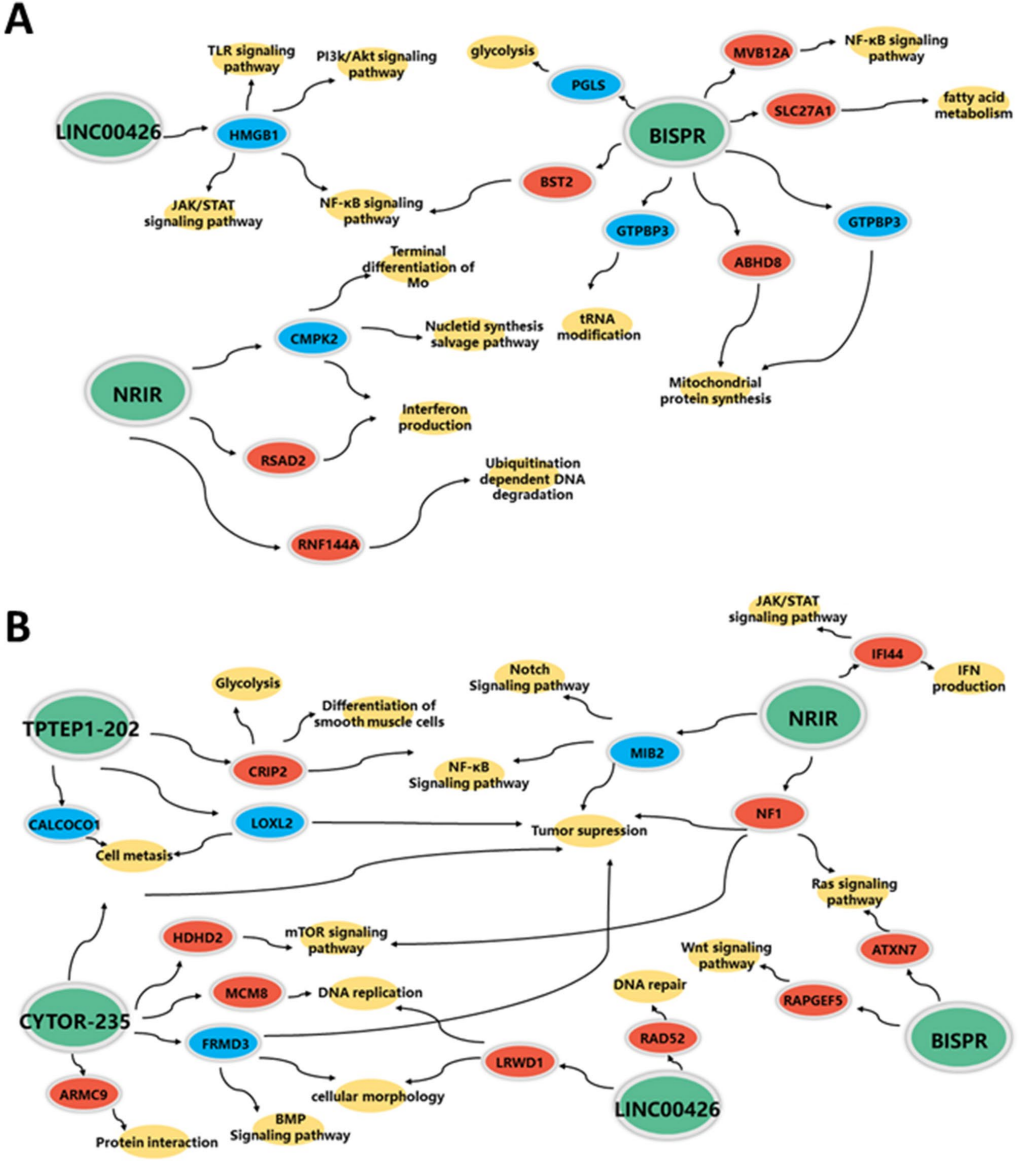
The gene pathway analysis of the co-expression and co-localization of mRNAs in this study. (**A**) The gene pathway network of the co-location mRNAs, green ellipses represented for LncRNA, red ellipses represent for the upstream mRNA, blue ellipses represent for the downstream mRNA and the yellow ellipses represent for the gene functions or signaling pathways. (**B**) The gene pathway network of the co-expression mRNAs, green ellipses represent for LncRNA, red/blue ellipses represent for the co-expression mRNA (up-regulated/down-regulated); yellow ellipses represent the gene functions or signaling pathways.

Tables 3 and 4 show the results of co-localization and co-expression analysis. LncRNA might regulate the adjacent protein-coding genes. Among the 22 co-localized mRNAs, HMGB1, RSAD2, CMPK2, MVB12A, and BST2 were reported to be strongly related to the development and differentiation of immune cells and regulation of the release of the inflammatory cytokines. PGLS, MRPL34, SLC27A1, and ABHD8 were reported to be significantly related to the mitochondrial function, glycolysis, and fatty acid metabolism. Other co-localized mRNAs were related to the degradation and modification of DNAs and RNAs. Among the 21 co-expressed mRNAs, IFI44, RAPGEF5, HDHD2, CRIP2, MIB2, and NF1 were strongly related to the immune-related signaling pathways and the release of the inflammatory cytokines. LOXL2, CALCOCO1, CRIP2, and HSD17B11 were associated with glycolysis, fatty acid metabolism, and cell metastasis. Other co-localized mRNAs were related to DNA repair, the regulation of tumor and cell morphogenesis. The gene network of the co-localized and co-expressed mRNAs are shown in Fig. 4. The differentially expressed LncRNAs might have been involved in the pathogenesis of pSS through interaction with co-expressed and co-localized mRNAs.

**Table 3:** The results of co-localization analysis between differentially expressed LncRNAs and mRNAs.

LncRNA gene name	P-value	Fold change	Regulation	Co-located mRNA name	Location
LINC00426	0.001	0.26	Down	KATNAL1	Upstream
				HMGB1	Downstream
				UBE2L5	Upstream
TPTEP1-202	0.002	0.34	Down	CCT8L2	Upstream
NRIR	0.003	5.28	Up	RSAD2	Upstream
				CMPK2	Downstream
				RNF144A	Upstream
BISPR	0.012	2.2	Up	PGLS	Upstream
				GTPBP3	Downstream
				MRPL34	Downstream
				ACO10319.2	Downstream
				PLVAP	Upstream
				CCDC194	Upstream
				AN08	Upstream
				SLC27A1	Upstream
				MVB12A	Upstream
				ABHD8	Upstream
				NXNL1	Downstream
				DDA1	Downstream
				BST2	Upstream
				TMEM221	Downstream

**Table 4:** The detailed results of the co-expression analysis between the differentially expressed LncRNAs and mRNAs.

LncRNA gene name	P-value	Fold change	Regulation	Correlated mRNA name	r	P-value
LINC00426	0.001	0.26	Down	RAD52	0.953	0.00002
				TPRG1	0.955	0.00002
				LOXL2	0.957	0.00001
				CALCOCO1	0.957	0.00001
				LRWD1	0.951	0.00002
TPTEP1-202	0.002	0.34	Down	CRIP2	0.961	0.00001
				ZNF286B	0.953	0.00002
CYTOR	0.002	1.64	Up	FRMD3	0.967	0.000005
				MCM8	0.954	0.00002
				HSD17B11	0.951	0.00002
				HDHD2	0.952	0.00002
				TEX46	0.950	0.00003
				GOLGA80	0.952	0.00002
				ARMC9	0.951	0.00002
NRIR	0.003	5.28	Up	RAPGEF5	0.953	0.00002
				IFI44	0.959	0.00001
				MIB2	0.961	0.00001
				FAM241B	0.963	0.000008
				NF1	0.961	0.000009
BISPR	0.012	2.2	Up	ATXN7	0.951	0.00002
				FAM149B1	0.954	0.00001

### The results of GO and KEGG analysis for the differentially expressed mRNAs

**Figure 5:**
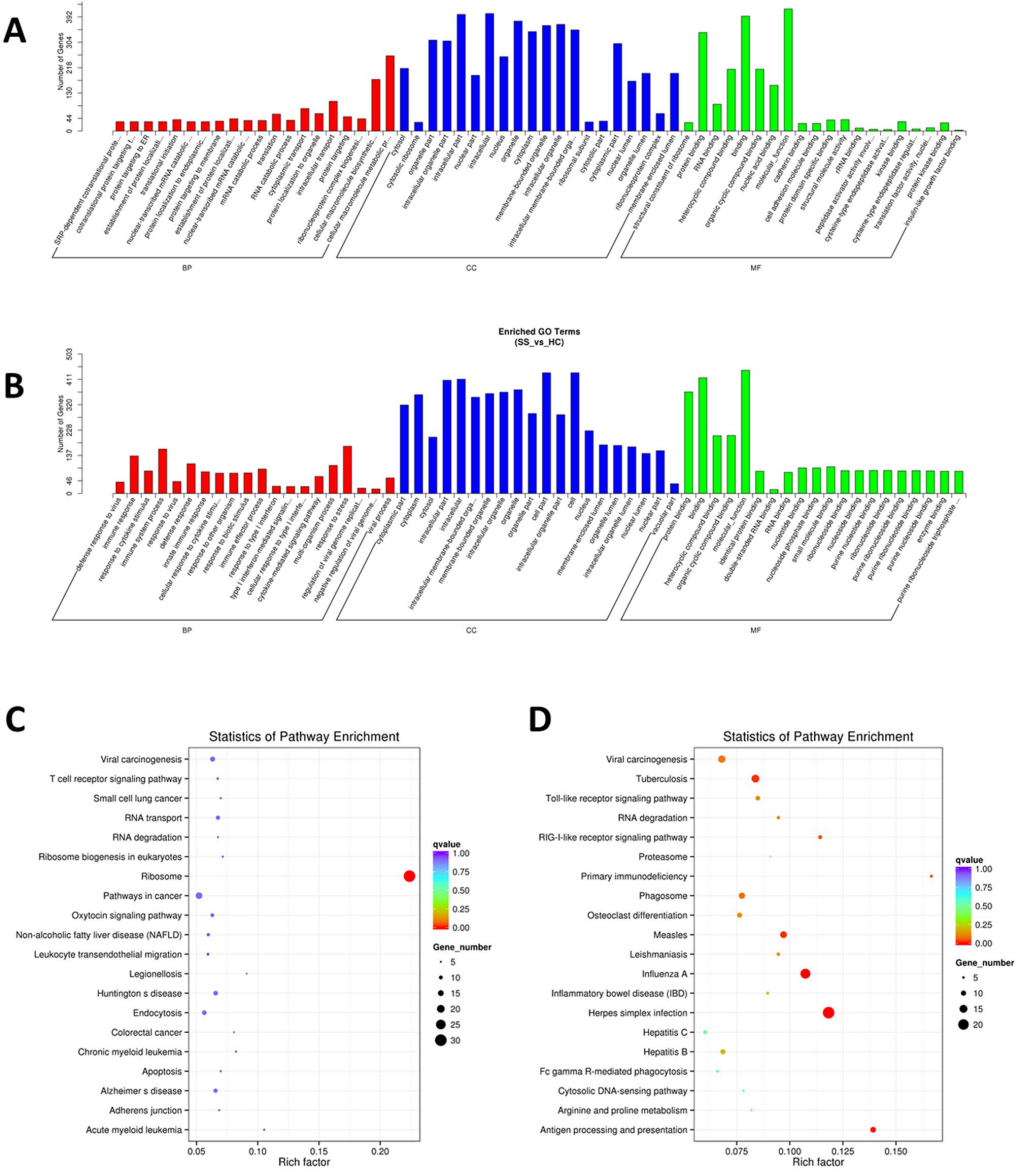
The results of GO and KEGG analysis of the differentially expressed mRNAs in the study. (**A**) The GO results for the down-regulated mRNAs. (**B**) The GO results for the up-regulated mRNAs. (**C**) The KEGG analysis results for the down-regulated mRNAs^21,22^. (**D**) The KEGG analysis results for the up-regulated mRNAs^21,22^.

GO and KEGG pathway analysis was mainly used to discover the potential pathogenic mechanism in pSS patients. Figure 5 shows the results of the Go and KEGG analysis. We found that the top significantly enriched biological process (BP) for the down-regulated mRNAs were cellular macromolecule metabolic process, cellular macromolecule biosynthetic process, intracellular transport, cytoplasmic transport, and translation while the top significantly enriched BP for the up-regulated mRNAs were immune system process, response to stress, immune response, defense response, and immune effector process. The most significantly enriched cellular component (CC) for the down-regulated mRNAs were intracellular parts, intracellular organelle, membrane-bounded organelle, and intercellular organelle. In case of the up-regulated mRNAs, it was cell part, cell, intracellular part, intercellular, and organelle. The significantly enriched molecular function (MF) for down-regulated mRNAs were molecular function, binding, protein binding, organic cyclic compound binding and heterocyclic compound binding. The same MFs were enriched for the up-regulated mRNAs. The results of KEGG pathway analysis for the differentially expressed mRNAs indicated that the down-regulated mRNAs in PBMCs of pSS patients were significantly enriched in ribosome, acute myeloid leukemia, legionellosis, chronic myeloid leukemia, and colorectal cancer; whereas the up-regulated mRNAs were significantly enriched in primary immunodeficiency, antigen processing and presentation, herpes simplex infection, RIG-I-like receptor signaling pathway, and influenza A, which were all closely related to the immunity.

## Discussion

With the development of the sequencing technology, LncRNAs have increasingly being found to be involved in various biological processes. Through its interaction with DNA, RNA, and proteins, LncRNAs could influence the activity of immune cells at transcriptional or post-transcriptional levels^23^. Nowadays, an increasing number of aberrantly expressed LncRNAs and their functions are being reported in the autoimmune diseases and infectious diseases^24,25^. Although the potential role of LncRNAs in pSS has been speculated previously, the detailed role of LncRNAs in the pathogenesis of pSS remains unknown. In this study, we used the RNA-seq technology to identify the differentially expressed LncRNAs and mRNAs in PBMCs of pSS patients, and to find out the potential function of these genes.

In this study, we enrolled 21 paired pSS patients and healthy controls. Through sequencing analysis we found that 44 out of 1772 LncRNAs and 1034 out of 15,424 mRNAs were differentially expressed in the PBMCs of pSS patients.

The up-regulated mRNAs were significantly enriched in the immune system process, response to stress, and immune response, indicating that a variety of immune-related genes were aberrantly up-regulated and participated in the pathogenesis of pSS. And the KEGG pathway analysis revealed that up-regulated mRNAs were enriched in primary immunodeficiency, antigen processing and presentation, herpes simplex infection, RIG-I-like receptor signaling pathway, and influenza A. Similar to other autoimmune diseases, pSS patients also had a variety of autoantigens (mainly SSA and SSB antigen), which could be presented to the T cells or be recognized by B cells to trigger the release of autoantibodies to promote disease progression^26,27^, and this attached importance to the enrichment function of antigen presentation, which indicated that LncRNAs were also involved in the production of pSS autoantibodies. Previous study of the LncRNA in the monor salivary glands of pSS patients also had the similar results^28^, and some immune-related signaling pathways like NF-κB signaling pathway, TNF signaling pathway, and natural killer cell mediated cytotoxicity were significantly enriched in the minor salivary gland of pSS patients. This indicated that LncRNAs participated in the immune disorder of pSS. Meanwhile, the analysis of the down-regulated mRNAs also enriched in immune function and metabolic function of PBMCs, indicating that LncRNAs were related to the regulation of the immunity and metastasis of PBMCs; thus, contributing to the pathogenesis of pSS.

Eleven selected LncRNAs were involved in the RT-qPCR validation stage. Among them, LINC00426, TPTEP1-202, CYTOR, NRIR, and BISPR were conformed to be aberrantly expressed. These differentially expressed LncRNAs were strongly related to the ESSDAI and serum IgG, CRP, and C4 (The correlation of CRP and C4 seemed to be affected by the particularly high outlier samples and were still needed the larger sample size study), which were closely correlated with disease activity of pSS. Thus, these differentially expressed LncRNAs were thought to affect pSS disease activity, which would be further verified in the subsequent experimental study. It was noted that the NRIR and BISPR were significantly up-regulated in the pSS patients as compared to the healthy controls. LncRNA-NRIR was located closely to the protein-coding interferon stimulated genes (ISGs), and CMPK2 was strongly correlated to the JAK-STAT signaling pathway, which acts as a negative regulator of IFN response^29^. It was reported that the knockdown of the LncRNA resulted in an increase in the Type I IFN-stimulated transcription. While BISPR was a BST2 (IFN-α stimulation) promotor-sharing LncRNA^30^ that was dependent on the JAK-STAT signaling pathway, overexpression of BISPR could up-regulate BST2 and induce the release of IFN-α. The activation of Type I IFN pathway has been found to play a vital role in the pathogenesis of pSS patients before^31,32^. Various factors like viral infection or immunity disorders can promote the release of type I IFN in pSS patients and activate the immune cells to release the inflammatory cytokines, antigen presentation, and production of autoantibodies, which was consistent with our LncRNA sequencing results. The differentially expressed LncRNA-NRIR and BISPR also attached importance to the Type I IFN signaling pathway in the pathogenesis of pSS. Wang et al. in their study found that LncRNA-TMEVPG1, which could regulate the release of IFN-γ, elevated in the pSS patients’ CD4+ T cells^33^ and were related to patients Th1 cells, ESR, serum IgG and anti-SSA antibody, this results just similar to the result of our study that the LncRNAs, especially the IFN related LncRNAs, were significantly differently expressed in the pSS patients PBMCs. While Some novel LncRNAs were also found differentially expressed in the sequencing study, like XLOC_167004, XLOC_102167, and XLOC_078623, their fold changes showed that they were highly expressed and their potential function could be verified in the future experiments.

LncRNAs could regulate the function of adjacent or correlated mRNAs. Therefore, in our study, we used the co-expression and co-localization analysis between the conformed differentially expressed LncRNAs and mRNAs to elaborate the potential mechanism of the LncRNAs in PBMCs of pSS patients. The results revealed that many immune-related genes were involved in PBMCs of pSS. HMGB1, which is located downstream of LINC00426, is a DNA-binding nuclear protein that has been implicated in some inflammatory disorders^34,35^. It could activate the innate immune cells (macrophages/monocytes) through interaction with TLR-2/4 and induce the release of cytokines like IL-8 through the NF-κB or MAPK signaling pathways. RASD2 (located upstream of NRIR) and IFI44 (correlated with NRIR) were reported to be the IFN inducers dependent on the JAK-STAT signaling pathway^36^, which can activate the innate immune cells and promote the release of IFN. CMPK2 (located downstream of NRIR) was thought to be related with the differentiation of monocytes and the release of I-IFN through the JAK-STAT signaling pathway^37^. BST2 (located upstream of BISPR) was reported to promote the innate immune release of inflammatory cytokines through the NF-κB signaling pathways^38^. NF1 (correlated with NRIR) could promote the development and differentiation of the immune cells through mTOR signaling pathway^39^. These immune cells, inflammatory cytokines, and signaling pathways were reported to be important in the pathogenesis of pSS. LOXL2 (correlated with LINC00426), CALCOCO1 (correlated with LINC00426), CRIP2 (correlated with TPTEP1-202), HSD17B11 (correlated with CYTOR), and SLC27A1 (upstream of BISPR) could regulate the metastasis of the PBMCs to regulate the corresponding cell function and the release of cytokines^40–44^, which could provide new insights into the pathogenesis of pSS. LncRNA-PVT1 participated in the regulation of cell metabolism through reprogramming glycolysis in CD4+ T cells to regulate immunity have been reported recently^45^, and similar to our results, LncRNAs regulating immunity through cell metabolism provided new direction for the pathogenesis of pSS. The results of the co-localization and co-expression analysis indicated that LncRNAs which could regulate the immune response of PBMCs were involved in the pSS disease progression. However, the mechanism of LncRNA still needs further studies, and animal models should also be used to further confirm the function of LncRNAs.

## Supplementary information

Supplementary Information.
