# Supplementary material for: LncRNA and mRNA expression profile of peripheral blood mononuclear cells in primary Sjögren’s syndrome patients
Source: Sci Rep. 2020 Nov 12;10:19629. doi: 10.1038/s41598-020-76701-2 (PMC7661519; doi:10.1038/s41598-020-76701-2)
Supplement: Supplementary file 1 — Supplementary Information. [file 41598_2020_76701_MOESM1_ESM.docx]

**LncRNA and mRNA expression profile of peripheral blood mononuclear cells in** **primary Sjögren’s** **syndrome patients**

**Authors**

Yu Peng^1,2*^, Xuan Luo^1,2*^, Yingying Chen^1,2^, Linyi Peng^1,2^, Chuiwen Deng^1,2^, Yunyun Fei^1,2#^, Wen Zhang^1,2#^, Yan Zhao^1,2#^

**Supplementary materials**

**Supplementary table 1. The details of primer pairs used in the analysis of the RT-qRCR in this study.**

| **LncRNA name** | **Forward primer (5’-3’)** | **Reverse primer (5’-3’)** |
| --- | --- | --- |
| LINC00426 | CACACAATGTTCTCATCGCCC | GGACAGTGACATCTCACTTCCCA |
| TPTEP1-202 | CCAGAAAGAAACTCAGCCCAC | TGTGAAGAGACCACCAAACAGG |
| CYTOR-235 | CCAATGAGAATGAAGGCTGAGACA | ACTGTGCTGTGAAGATCTGAAGAC |
| RN7SL834P | CCAGTTACTCAGAAGACAGAAGCA | GCATGGATATCTCATTGGCATAG |
| NRIR | CCATTCATAACCTCATAAACCACC | ACCATCTCACAATGTGCCCA |
| DTX2P1 | TCACTGCCAGAGGGTTTCCC | ACGGGCAGCGTCATGTAGT |
| BISPR | GTACATGCCTGTAATCCCAACACTT | GGAAGGATTTTGTTGCTCACACTAG |
| RN7SL141P | TCGCACTAAGTTCAGCATTAATGG | TGATGGCTATTCATGGGCATGT |
| LINC01550 | TACCGAGCTTTACAGCCATATTGA | CAGTGTTATTTACCAGCAGGAAAAG |
| SNHG8 | GCCTTTCTTCCAAATCATCAGC | GCAGTAGAGGATCAGGAATGGTG |
| LOC105373098 | GTCATGTTCCTTACTAACAGCACGT | GCTCTTTCAGTCAGGTGTTCCC |
